# Exposure to Mitochondrial Toxins: An In Vitro Study of Energy Depletion and Oxidative Stress in Driving Dopaminergic Neuronal Death in MN9D Cells

**DOI:** 10.3390/toxics13080637

**Published:** 2025-07-29

**Authors:** Oluwatosin Adefunke Adetuyi, Kandatege Wimalasena

**Affiliations:** Department of Chemistry and Biochemistry, Wichita State University, Wichita, KS 67260, USA; kandatege.wimalasena@wichita.edu

**Keywords:** mitochondrial dysfunction, oxidative stress, rotenone, antimycin A, dopamine metabolism, neuromelanin, Parkinson’s disease, neurodegeneration

## Abstract

Mitochondrial dysfunction is a key contributor to neurodegeneration, particularly in Parkinson’s disease (PD), where dopaminergic neurons being highly metabolically active are vulnerable to oxidative stress and bioenergetic failure. In this study, we investigate the effects of rotenone, a Complex I inhibitor, and antimycin A, a Complex III inhibitor, on mitochondrial function in MN9D dopaminergic neuronal cells. Cells were treated with rotenone (1.5 µM) or antimycin A (10 µM) for one hour, and key biochemical parameters were assessed, including ATP levels, reactive oxygen species (ROS) production, dopamine metabolism, and neuromelanin formation. Our results indicate significant ATP depletion and ROS accumulation following treatment with both inhibitors, with antimycin A inducing a more pronounced oxidative stress response. Dysregulation of dopamine biosynthesis differed mechanistically from vesicular monoamine transporter (VMAT2) inhibition by tetrabenazine, suggesting alternative pathways of catecholamine disruption. Additionally, oxidative stress led to increased neuromelanin accumulation, indicating a possible adaptive response to mitochondrial dysfunction. These findings provide insights into the cellular mechanisms underlying dopaminergic neurotoxicity and highlight mitochondrial electron transport chain inhibition as a key driver of PD pathogenesis. Future research should explore therapeutic strategies aimed at enhancing mitochondrial function to mitigate neurodegenerative progression.

## 1. Introduction

Parkinson’s disease (PD) is a progressive neurodegenerative disorder characterized by the selective degeneration of dopaminergic neurons in the substantia nigra pars compacta (SNpc), leading to motor dysfunction, cognitive decline, and other debilitating symptoms. While genetic mutations account for several forms of early-onset PD [1], exposure to environmental toxins has been increasingly recognized as significant risk factors [2]. For example, a number of agricultural pesticides [3], industrial pollutants [4], and other environmental toxicants [5] have been implicated in PD etiology, yet the precise molecular mechanisms by which these toxins contribute to dopaminergic degeneration remain incompletely understood [6]. Dopaminergic neurons in SNpc exhibit a high metabolic energy demand due to their continuous synaptic activity (pace-keeping activity) [7] and other special characteristics [8]. Thus, their survival is highly dependent on the efficient mitochondrial oxidative phosphorylation for energy generation [8] highly susceptible to mitochondrial dysfunction, which is proposed to be a key pathological feature in PD.

Mitochondrial dysfunction associated with impaired oxidative phosphorylation causing increased reactive oxygen species (ROS) production, and disrupted mitophagy [9] and dysregulation of catecholamine homeostasis have been previously demonstrated in dopaminergic neurons [10,11,12]. In addition, dysregulation of DA metabolism under these conditions could lead to accumulation of cytosolic DA, leading to its autoxidation followed by polymerization to produce neuromelanin [13,14]. While neuromelanin may act as an antioxidant at low concentrations by sequestering redox-active metals, excessive accumulation under oxidative conditions can lead to toxic neuromelanin aggregation, further promoting neuronal degeneration. Excessive accumulation of neuromelanin can also exacerbate neurotoxicity by releasing the bound toxic metal and other toxic substances [15]. Observed increase in the sensitivity of neuromelanin containing neurons in SNpc to PD could be due to the excessive accumulation of neuromelanin as hypothesized in our recent publication [16]. In good agreement with these proposals, recent studies suggest that neuromelanin formation is upregulated under oxidative stress conditions, initially providing a potential defense mechanism against mitochondrial dysfunction [15] while excessive accumulation could lead to increased oxidative stress causing toxic neuromelanin aggregation, further promoting neuronal degeneration.

Our recent hypothesis proposed a mechanistic framework in which environmental toxins affect mitochondrial energy production pathways, leading to metabolic bioenergetic failure, dysregulation of DA metabolism and increased oxidative stress, ultimately causing degeneration of dopaminergic neurons in PD [16]. Although this hypothesis is consistent with reports demonstrating mitochondrial Complex I & III inhibition by toxins such as rotenone and paraquat, causes dopaminergic degeneration [17,18], yet conflicting views exist regarding whether mitochondrial damage is a primary event or a consequence of broader cellular dysfunction, including lysosomal and proteasomal degradation impairments [19,20] under these conditions. To resolve these possibilities, the present study aims to experimentally validate key aspects of our hypothesis by investigating the effects of rotenone and antimycin A—known inhibitors of mitochondrial Complex I and III, respectively—on dopaminergic MN9D cells in vitro. We have examined the effects of these inhibitors on mitochondrial bioenergetics, oxidative stress markers, dopamine metabolism, and neuromelanin accumulation, etc., to determine the extent to which mitochondrial inhibition of energy production contributes to PD-related neuropathology. The findings in the present manuscript will not only provide mechanistic insights into the mitochondrial toxin-mediated dopaminergic neuronal death but also help reconcile divergent hypotheses regarding the primary drivers of toxin-induced neurodegeneration. Understanding these mechanisms is critical for advancing PD research, as it may inform therapeutic strategies aimed at enhancing mitochondrial function or mitigating environmental toxin exposure. Given the ongoing debate over whether mitochondrial dysfunction initiates or exacerbates PD pathology, this study serves as an important step towards resolving this question. Our findings may also provide a foundation for future studies exploring neuroprotective interventions targeting mitochondrial quality control and oxidative stress pathways.

## 2. Materials and Methods

### 2.1. Chemicals and Reagents

All chemicals and solvents used in this study were purchased from commercial vendors and were of the highest available purity. Krebs-Ringer buffer supplemented with HEPES (KRB-HEPES) was composed of 125 mM NaCl, 5.34 mM KCl, 0.81 mM MgSO_4_, 1.3 mM CaCl_2_, 0.77 mM NaH_2_PO_4_, 25 mM HEPES, and 5.55 mM glucose, pH adjusted to 7.4. Dulbecco’s Modified Eagle Medium (DMEM) contained 109.5 mM NaCl, 5.34 mM KCl, 0.81 mM MgSO_4_, 1.8 mM CaCl_2_, 0.77 mM NaH_2_PO_4_, 44 mM NaHCO_3_, and 5.55 mM glucose. Stock solutions of rotenone, 4′,6-diamidino-2-phenylindole, dilactate (DAPI), and 2′,7′-dichlorofluorescin diacetate (DCFH-DA) and Antimycin A were prepared in dimethyl sulfoxide and stored at −20 °C until use. In all experiments, final dimethyl sulfoxide concentration was kept to a minimum, usually <0.05% *v*/*v*. For all cell-based experiments, working solutions were freshly prepared by diluting the stock solutions in KRB-HEPES buffer (pH 7.5), ensuring that the final DMSO concentration did not exceed 0.05% *v*/*v*. This low level of DMSO was chosen to avoid any cytotoxic or confounding effects, as such concentrations are generally considered negligible and comparable to what cell lines are routinely exposed to during cryopreservation [21,22,23,24]. Control treatments were conducted using KRB-HEPES buffer alone, to match the physiological conditions of the treated cells without introducing variable solvent effects. Tetrabenazine was directly dissolved in KRB-HEPES buffer (pH 7.5) to prepare both stock and working solutions, as it was readily soluble in aqueous conditions. All treatment solutions were freshly prepared before use. Fontana mason stain kit was also obtained from Sigma-Aldrich, St. Louis, MO, USA (Catalog # HT200).

### 2.2. Cell Lines

The MN9D (mouse hybridoma cell line) used in this study was kindly provided by Dr. Alfred Heller, University of Chicago while we obtained the human hepatocellular liver carcinoma (HepG2) cell line from Dr. Tom Wiese (Fort Hays University, Hays, KS, USA).

### 2.3. Instrumentation

Fluorescence imaging for DAPI-stained and DCFH-DA-loaded cells was performed using a Nikon ECLIPSE Ti inverted microscope equipped with a 40× Fluoro objective (Nikon Instruments Inc., Melville, NY, USA). ROS fluorescence (Ex/Em 488/524 nm) was detected using the same microscope with consistent imaging parameters across treatment groups.

### 2.4. Cell Culture

MN9D dopaminergic neuronal cells and HepG2 liver cells were grown in 100 mm^2^ Falcon tissue culture plates containing DMEM with 4.5 g/L glucose, supplemented with 10% fetal bovine serum (FBS). Cells were cultured at 37 °C in a humidified atmosphere containing 7% CO_2_. Cells were seeded into 12-well plates, 96-well plates, or glass-bottom dishes depending on the experiment, and allowed to grow to approximately 70–80% confluency before treatment.

### 2.5. Measurement of Cell Viability

Cell viabilities were determined by the MTT [3-(4,5-dimethylthiazol-2-yl)-2,5-diphenyltetrazolium bromide)] cell viability assay. Cells were seeded on 96-well plates and allowed to grow to about 70–80% confluence. After media removal, cells were exposed to various toxins in KRB-HEPES buffer and incubated for 12 h at 37 °C. After treatment, 10 µL of 5 mg/mL MTT solution was added to each well and incubated for an additional 2 h. Formazan crystals formed were solubilized with 210 µL of a detergent solution (50% dimethylformamide, 20% SDS) and incubated for 4 h at 37 °C. Solubilized formazan was quantified by measuring the difference in the absorbance at 570 and 650 nm and this was used to estimate cell viability. Cell viability result was expressed as % viability of toxin-treated cells relative to control cells with no toxin in the incubation media.

### 2.6. Measurement of Intracellular ATP Levels

Intracellular ATP levels were measured using ATP Colorimetric and Fluorometric Assay kit obtained from Tribioscience, Sunnyvale, CA, USA (Catalog # TBS2010). Tribioscience’s ATP assay kit utilizes the phosphorylation of glycerol by ATP to generate glycerol-3-phosphate whose absorbance can be measured at 570 nm. Briefly, cells were seeded in a 12 well plate and allowed to grow to about 70–80% confluence, and the growth media were replaced with KRB-HEPES containing a desired concentration of toxins and incubated for 1 h. Cells were lysed in 100 μL of assay buffer. Lysed cells were added to 96 well plates and 90 μL of ATP reaction mix (containing Assay buffer, probe, and substrate (all provided by the vendor)) were added to each well. Absorbance of the reaction product (Glycerol-3-phosphate) was measured at 570 nm. The absorbance readings were converted to the corresponding ATP concentration using a standard curve constructed employing standard ATP provided by Tribioscience.

### 2.7. Measurement of Catecholamine Levels

MN9D cells were plated in 12-well plates and treated with toxins for 1 h at 37 °C in KRB-HEPES buffer. Following treatment, cells were rinsed, collected in 1 mL of cold buffer, and a 50 µL aliquot was taken for protein estimation. The remaining suspension was centrifuged at 6000 rpm for 3 min. Pellets were resuspended in 75 µL of 0.1 M perchloric acid (HClO_4_), and then centrifuged at 13,200 rpm for 10 min. Supernatants were analyzed for DOPA and dopamine levels using high-performance liquid chromatography with electrochemical detection (HPLC-EC) [25]. Quantification was performed using standard calibration curves constructed using commercial standards, and the quantification results were normalized to protein concentration (nmol/mg protein). All experiments were repeated three times.

### 2.8. Measurement of Reactive Oxygen Species (ROS)

To measure intracellular ROS levels, cells seeded in 12-well plates were first incubated with 10 µM DCFH-DA in KRB-HEPES for 1 h. After washing, cells were treated with toxins in the same buffer for an additional 1 h at 37 °C. Cells were then lysed in 0.1 M Tris-HCl (pH 7.5) containing 1% Triton X-100. Lysates were centrifuged to remove debris, and the resulting supernatants containing ROS-oxidized DCFH-DA product, 2′,7′-dichlorofluorescein were analyzed for fluorescence at Ex/Em 504/526 nm to determine ROS production. Results were normalized to total protein of each sample. ROS accumulation in live cells was also visualized under a fluorescence microscope (Ex/Em 488/524 nm) following identical treatment and DCFH-DA loading.

### 2.9. Measurement of Neuromelanin Levels

Cells grown in 6-well plates were rinsed with KRB-HEPES pH 7.5 buffer and centrifuged at 900 rpm for 2 min at 37 °C. The pellet was washed with KRB-HEPES pH 7.5 buffer and centrifuged at 10,000 G for 15 min at RT. 1.5 mL of buffer containing 5 mg/mL SDS in 75 mM Tris pH 7.5 with 0.4 mg/mL proteinase K (Sigma Aldrich, St. Louis, MO, USA. Catalog # P2308) was added. Samples were sonicated and incubated for 3 h at 37 °C in a shaking water bath. The samples were then centrifuged at 10,000× *g* at RT for 30 min. The pellet was washed with 1.5 mL 0.9% NaCl and 1.5 mL Millipore water. The neuromelanin residue obtained was dissolved in 1 mL of 1M NaOH at 80 °C for 1 h. The mixture was centrifuged, and the absorbance of supernatant was measured at 420 nm. The absorbance readings were converted to the corresponding Neuromelanin concentrations using a standard curve constructed using standard melanin (Sigma Aldrich, St. Louis, MO, USA. Catalog #M0418). The protein concentration of each sample was determined by the Bradford protein assay. The data were normalized to the protein content of individual samples.

### 2.10. Fontana Masson Staining

Microscopic visualization of Neuromelanin production in toxin-treated MN9D cells was carried out using the Fontana Masson Stain kit obtained from Sigma-Aldrich according to the procedure outlined in the assay kit. Briefly, cells were grown in microscope plates and treated with toxins for 1 h. Cells were then incubated in freshly prepared ammoniacal silver solution for 1 h before rinsing several times with distilled water. Cells were then briefly incubated in Gold Chloride solution, rinsed several times with changes in distilled water, followed by incubation in sodium thiosulfate for 2 min. The cells were rinsed with distilled water and then counterstained with Nuclear Fast Red Solution, then rinsed in distilled water before imaging using a Light Microscope.

### 2.11. Western Blot Analysis

The expression levels of Tyrosine Hydroxylase (TH), Dopa Decarboxylase (DDC), Dopamine β-Hydroxylase (DBH), Glutathione S-transferase Mu 2 (GSTM2), and NADPH Quinone Oxidoreductase 1 (NQO1) in MN9D cells were analyzed by Western blot using polyclonal antibodies from commercial vendors. MN9D cells were cultured in 12-well plates and grown to approximately 70–80% confluency under standard conditions. After two washes with phosphate-buffered saline (PBS), the cells were treated with toxins for 1 h. Following treatment, cells were washed again with PBS and collected via low-speed centrifugation.

Cell pellets were lysed in a buffer containing 50 mM Tris-HCl, 150 mM NaCl, and 1% Triton X-100, supplemented with protease and phosphatase inhibitors (as per manufacturer instructions; Sigma-Aldrich, St. Louis, MO, USA and Thermo Fisher, Waltham, MA, USA, respectively). Lysis was performed for 30 min at 4 °C. The resulting lysates were centrifuged at 13,200 rpm for 10 min, and the supernatants were collected for analysis. Protein concentrations were measured using the Bradford assay (Bio-Rad, Hercules, CA, USA) with bovine serum albumin as a standard. Equal amounts of protein were loaded across all samples.

For electrophoresis, 100 µg of each protein sample was denatured at 94 °C in Laemmli buffer for 5 min, then resolved on an 8.5% SDS-PAGE gel. Proteins were transferred onto 0.2 µm PVDF membranes (Bio-Rad) using standard transfer protocols. Membranes were blocked for 1 h at room temperature in TBS with 0.1% Tween-20 (TBST) containing 5% non-fat dry milk, then incubated overnight at 4 °C with primary antibodies: rabbit anti-TH (PhosphoSolutions, Aurora, CO, USA; 1:1000), and rabbit antibodies against DBH, DDC, GSTM2, and NQO1 (Assay Biotech, San Jose, CA, USA; each at 1:500). Mouse anti-β-actin (Invitrogen, Carlsbad, CA, USA; 1:5000) was used as a loading control.

After washing in TBST, membranes were incubated with HRP-conjugated secondary antibodies (Bio-Rad; 1:5000) prepared in blocking buffer. Detection was carried out using an HRP substrate reagent kit (Bio-Rad), and chemiluminescent signals were visualized and quantified using a Gel Logic 100 imaging system (Bio-Rad, Hercules, CA, USA). Quantitative analysis was performed to assess relative protein expression across treatment conditions.

### 2.12. Protein Determination

Protein content in cell lysates was determined using the Bradford assay. A 50 µL sample in KRB-HEPES was mixed with 950 µL of Bradford reagent and incubated at room temperature for 10 min. Absorbance at 595 nm was recorded and protein concentrations were calculated from a bovine serum albumin (BSA) standard curve.

### 2.13. Data Analyses

Data analyses were carried out using Graphpad prism 10 (GraphPad software, Inc., Boston, MA, USA). To account for minor variations in color development during the MTT assay for cell viability, absorbance values were expressed as a percentage relative to control samples, which underwent identical treatment excluding toxin exposure. Data represent the mean ± standard deviation (SD) from three to eight replicates. Error bars indicate the SD from the mean. For quantitative analysis of cellular uptake, ATP levels, and ROS production, all measurements were normalized to the protein content of each sample to account for differences in cell density across experiments. Statistical significance for pairwise comparisons was determined using ordinary one-way ANOVA, with a *p*-value of < 0.0001 considered to be statistically significant.

### 2.14. Technical Statement

Due to the high toxicity and potential health risks associated with rotenone and antimycin A, all handling procedures followed established safety guidelines and were conducted with extreme care.

## 3. Results

### 3.1. Rotenone and Antimycin A Significantly Reduced ATP Production in MN9D Cells

**Figure 1:**
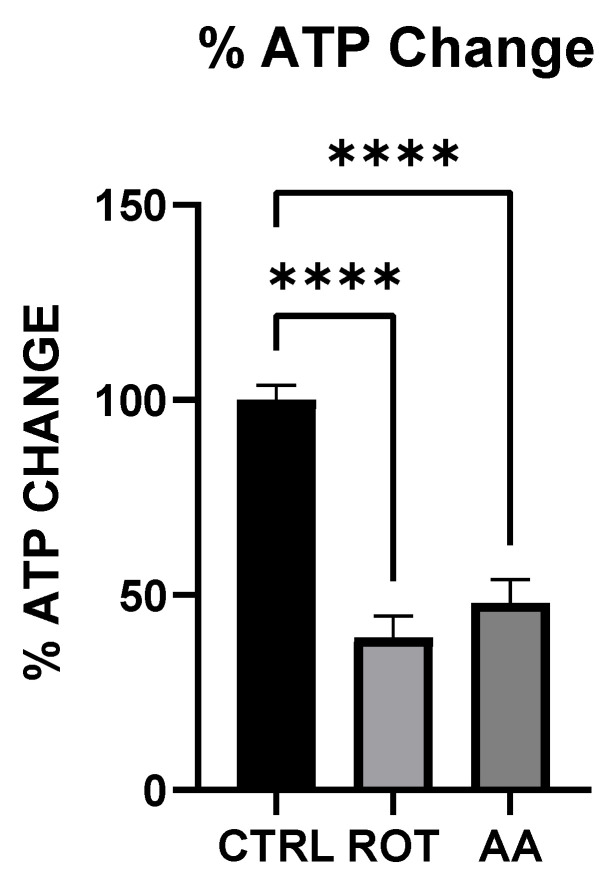
Effect of Toxins treatment on ATP level. Rotenone and Antimycin A significantly reduced ATP production in MN9D cells (data are represented as mean ± SD (*n* = 3), **** *p* < 0.0001 indicates statistical difference from control by one-way ANOVA).

Figure 1 shows the ATP level in MN9D cells after exposure to toxins. To evaluate the effect of mitochondrial toxins on cellular energy production, MN9D cells were incubated in Control (KRB-HEPES pH7.5 buffer), Rotenone (1.5 µM) and Antimycin A (10 µM) for 1 h. ATP levels were quantified using a colorimetric and fluorometric-based assay. Rotenone and Antimycin A significantly reduced ATP production in MN9D cells.

### 3.2. Rotenone and Antimycin A Disrupt Catecholamine Biosynthesis

**Figure 2:**
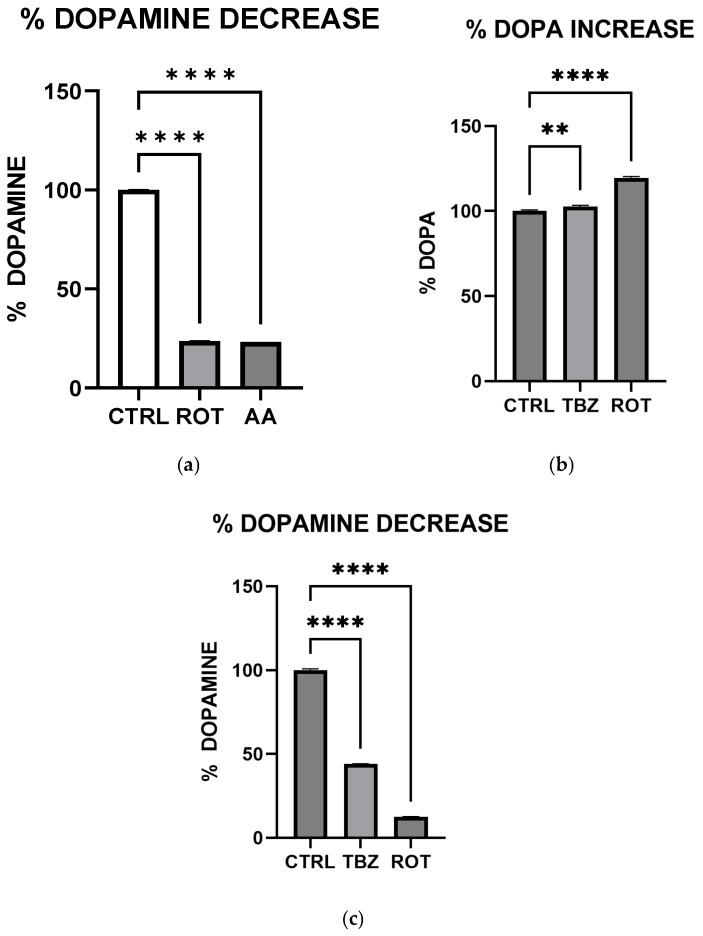
Rotenone and Antimycin A disrupt catecholamine biosynthesis. (**a**) Rotenone and Antimycin A effect on Dopamine biosynthesis. (**b**) Rotenone and Tetrabenazine effect on DOPA level in MN9D cells. (**c**) Rotenone and Tetrabenazine effect on Dopamine biosynthesis in MN9D cells (Data are represented as mean ± SD (*n* = 3), ** indicates difference from control and **** *p* < 0.0001 indicates statistical difference from control by one-way ANOVA).

To evaluate the effect of mitochondrial toxins on catecholamine biosynthesis, MN9D cells were incubated in Control (KRB-HEPES pH 7.5 buffer), Rotenone (1.5 µM), Antimycin A (10 µM) and Tetrabenazine (1 µM) for 1 h. We performed high-performance liquid chromatography to observe DOPA and Dopamine levels. HPLC analysis showed that dopamine levels were significantly reduced in rotenone-, antimycin A- (Figure 2a) and tetrabenazine (Figure 2c)-treated cells when compared to the control.

### 3.3. TH Protein Expression in Dopaminergic Neurons

**Figure 3:**
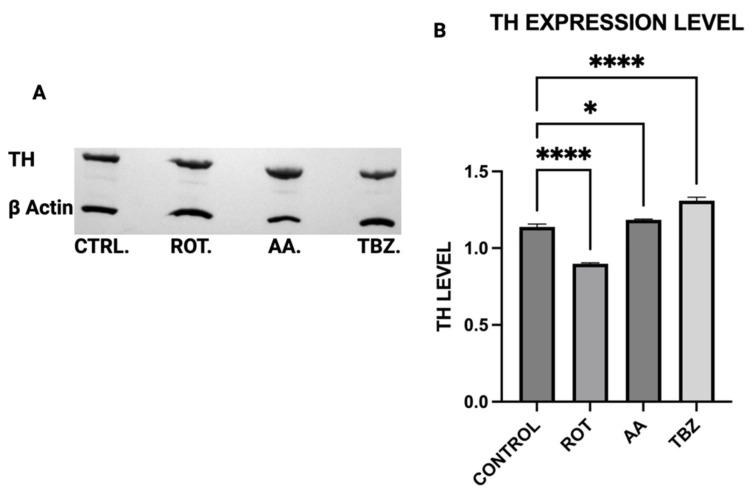
TH protein expression in dopaminergic neurons. (**A**) Representative Western blot of TH expression. (**B**) Quantification of Western blot. (Data are represented as mean ± SD (*n* = 3), * indicates difference from control **** *p* < 0.0001 indicates statistical difference from control by one-way ANOVA).

To determine the effects of mitochondrial toxins on dopamine biosynthesis at the enzymatic level, Tyrosine Hydroxylase expression in MN9D cells was assessed by Western blot analysis. MN9D cells were incubated in Control (KRB-HEPES pH 7.5 buffer), Rotenone (1.5 µM), Antimycin A (10 µM) and Tetrabenazine (1 µM) for 1 h. Quantification of Western blot showed reduced TH expression in Rotenone-treated group (Figure 3A,B).

### 3.4. DDC Protein Expression in Dopaminergic Neurons

**Figure 4:**
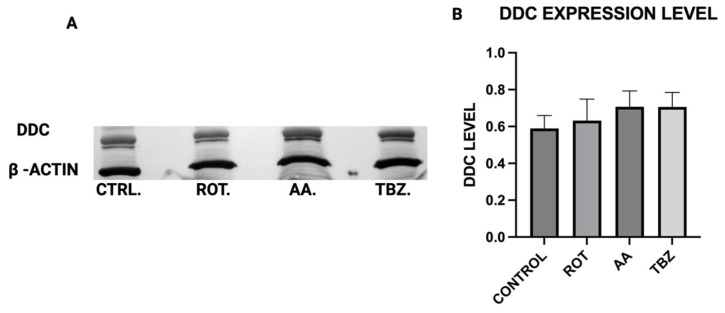
DDC protein expression in dopaminergic neurons. (**A**) Representative Western blot of DDC expression (**B**) Quantification of Western blot (Data are represented as mean ± SD (*n* = 3).

MN9D cells were incubated in Control (KRB-HEPES pH7.5 buffer), Rotenone (1.5 µM), Antimycin A (10 µM) and Tetrabenazine (1 µM) for 1 h, DDC expression level was assessed by Western blot analysis to determine whether mitochondrial toxins affect dopamine biosynthesis at the enzymatic level (Figure 4A). The quantification of Western blot showed that DDC expression was not different across all groups (Figure 4B).

### 3.5. DBH Protein Expression in Dopaminergic Neurons

**Figure 5:**
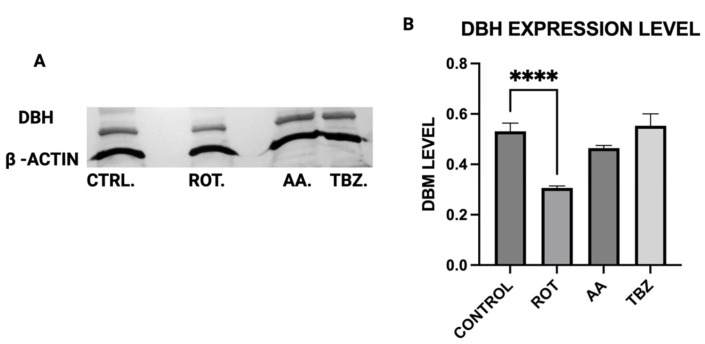
DBH protein expression in dopaminergic neurons. (**A**) Representative Western blot of DBH expression (**B**) Quantification of Western blot. (Data are represented as mean ± SD (*n* = 3), **** *p* < 0.0001 indicates statistical difference from control by one-way ANOVA).

MN9D cells were incubated in Control (KRB-HEPES pH7.5 buffer), Rotenone (1.5 µM), Antimycin A (10 µM) and Tetrabenazine (1 µM) for 1 h, DBH protein expression was assessed by Western blot analysis to determine whether mitochondrial toxins affect dopamine biosynthesis at the enzymatic level (Figure 5A). Quantification of Western blot showed reduced DBH expression in Rotenone-treated group (Figure 5B).

### 3.6. Toxins Treatment Reduced Cell Viability in MN9D Cells

**Figure 6:**
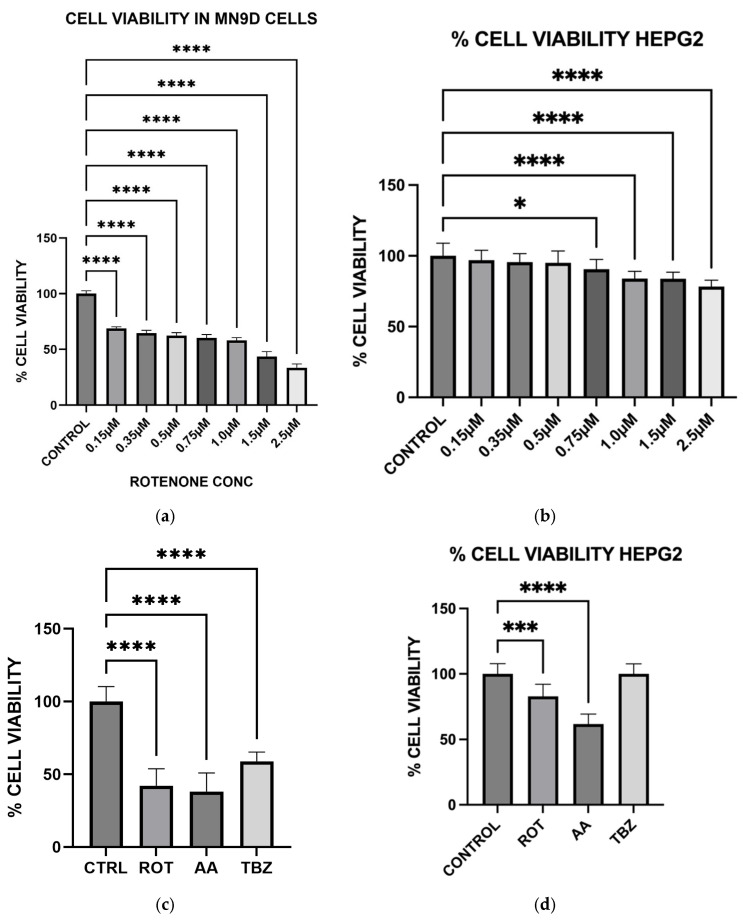
Effect of Toxins treatment on cell viability in MN9D and HEPG2 cells. (**a**) Rotenone effects on cell viability in MN9D cells at varying doses. (**b**) Rotenone effect on cell viability in HepG2 cells at varying doses. (**c**) When MN9D cells were exposed to all three compounds, both rotenone and antimycin A significantly reduced cell viability. (**d**) Cell viability In HepG2 cells following toxins treatment. (Data are represented as mean ± SD (*n* = 8), * and *** indicates difference from control, **** *p* < 0.0001 indicates statistical difference from control by one-way ANOVA).

To assess the cytotoxic effect of mitochondrial toxins, we evaluated cell viability in MN9D dopaminergic neurons and HepG2 liver cells using the MTT assay. MN9D cells were treated with increasing concentrations of rotenone and with single doses of rotenone (1.5 µM), antimycin A (10 µM), or tetrabenazine (1 µM) for 1 h. HepG2 cells were also subjected to identical treatments to investigate specificity of toxins to neuronal cells. In MN9D cells, rotenone significantly reduced cell viability in a dose-dependent manner, confirming its potent neurotoxic effect associated with Complex I inhibition (Figure 6a). In HepG2 cells, rotenone had no significant effect on cell viability at the tested concentration, suggesting selective vulnerability of dopaminergic neurons (Figure 6b). When MN9D cells were exposed to all three compounds, both rotenone and antimycin A significantly reduced cell viability compared to control, while tetrabenazine had no significant effect (Figure 6c). In HepG2 cells, antimycin A caused a marked decrease in viability, indicating that Complex III inhibition is broadly cytotoxic, though rotenone’s effect remained negligible in these non-neuronal cells (Figure 6d).

### 3.7. Effect of Toxins on Apoptotic Chromatin Condensation in MN9D Cells (DAPI Staining)

**Figure 7:**
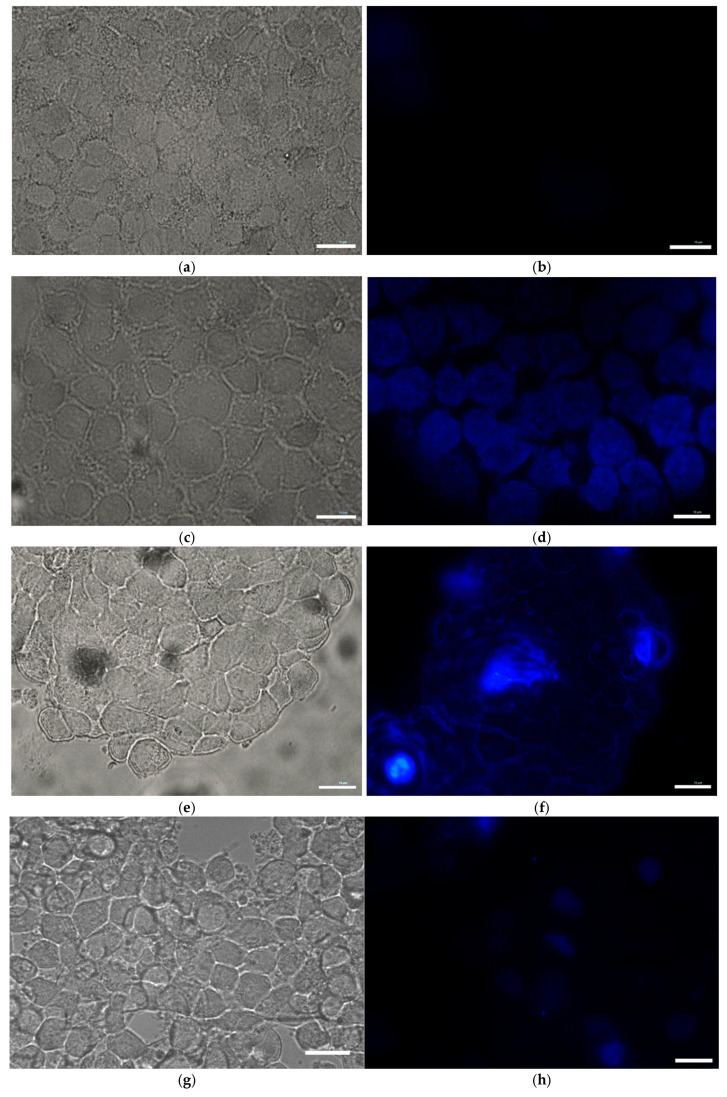
Effect of toxins on Apoptotic chromatin condensation in MN9D cells (DAPI staining). Representative images are shown for each treatment group: (**a**,**b**) control, (**c**,**d**) rotenone, (**e**,**f**) antimycin A, and (**g**,**h**) tetrabenazine. Scale bar: 10 µm.

To assess nuclear morphology and potential chromatin condensation characteristic of apoptosis, MN9D cells were treated for 1 h with either control buffer (KRB-HEPES pH 7.5), rotenone (1.5 µM), antimycin A (10 µM), or tetrabenazine (1 µM), followed by staining with 4′,6-diamidino-2-phenylindole (DAPI). In the control group (Figure 7a,b), nuclei appeared uniformly round with consistent DAPI staining intensity, indicating intact nuclear architecture. In contrast, rotenone Figure 7c,d), Antimycin A (Figure 7e,f)- and tetrabenazine (Figure 7g,h)-treated cells exhibited clear signs of chromatin condensation and nuclear fragmentation, hallmarks of apoptotic cell death.

### 3.8. Toxins Treatment Increased Oxidative Stress Level in MN9D Cells

**Figure 8:**
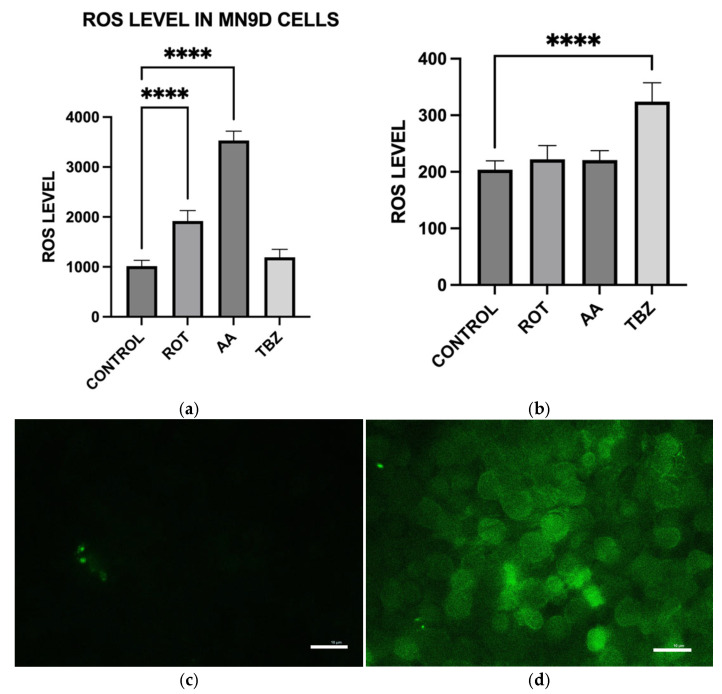

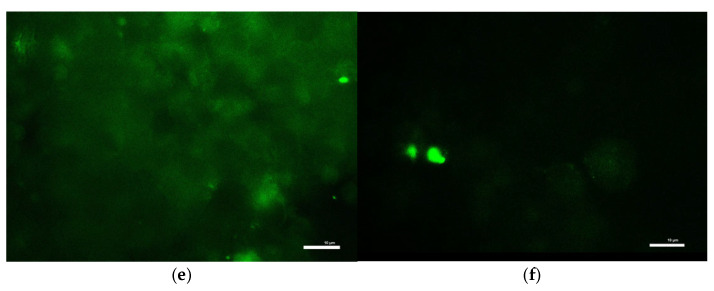
Effect of toxins treatment on oxidative stress level in MN9D and HEPG2 cells. (**a**) ROS levels in MN9D cells (**b**) ROS levels in HepG2 cells. (**c**–**f**) Representative fluorescence images showing DCF-DA staining of MN9D cells: (**c**) control, (**d**) rotenone-treated, (**e**) antimycin A-treated, and (**f**) tetrabenazine-treated. (Data are represented as mean ± SD (*n* = 8), **** *p* < 0.0001 indicates statistical difference from control by one-way ANOVA, ns indicates not statistically significant when compared to the control by one-way ANOVA). Scale bar: 10 µm.

To evaluate the oxidative stress response following mitochondrial toxin exposure, intracellular reactive oxygen species (ROS) levels were quantified in MN9D dopaminergic neurons and HepG2 liver cells using the DCF-DA fluorescence assay after a 1 h treatment with control buffer (KRB-HEPES pH 7.5), rotenone (1.5 µM), antimycin A (10 µM), or tetrabenazine (1 µM). In MN9D cells, both rotenone and antimycin A significantly elevated ROS levels compared to control (Figure 8a). Notably, antimycin A induced an increase, exceeding a three-fold rise in fluorescence intensity, indicating huge oxidative stress. Tetrabenazine did not significantly alter ROS levels, suggesting that VMAT2 inhibition does not contribute to oxidative stress in this model (Figure 8a).

In HepG2 cells, neither rotenone nor antimycin A caused a statistically significant increase in ROS levels, highlighting the relative resistance of these non-neuronal cells to mitochondrial stress compared to dopaminergic neurons (Figure 8b). Representative fluorescence images showing DCF-DA staining of MN9D cells displayed enhanced green fluorescence in the rotenone and antimycin A groups, reflecting increased ROS accumulation which is consistent with the quantitative results (Figure 8c–f).

### 3.9. Exposure to Toxins Increased Neuromelanin Production in MN9D Cells

**Figure 9:**
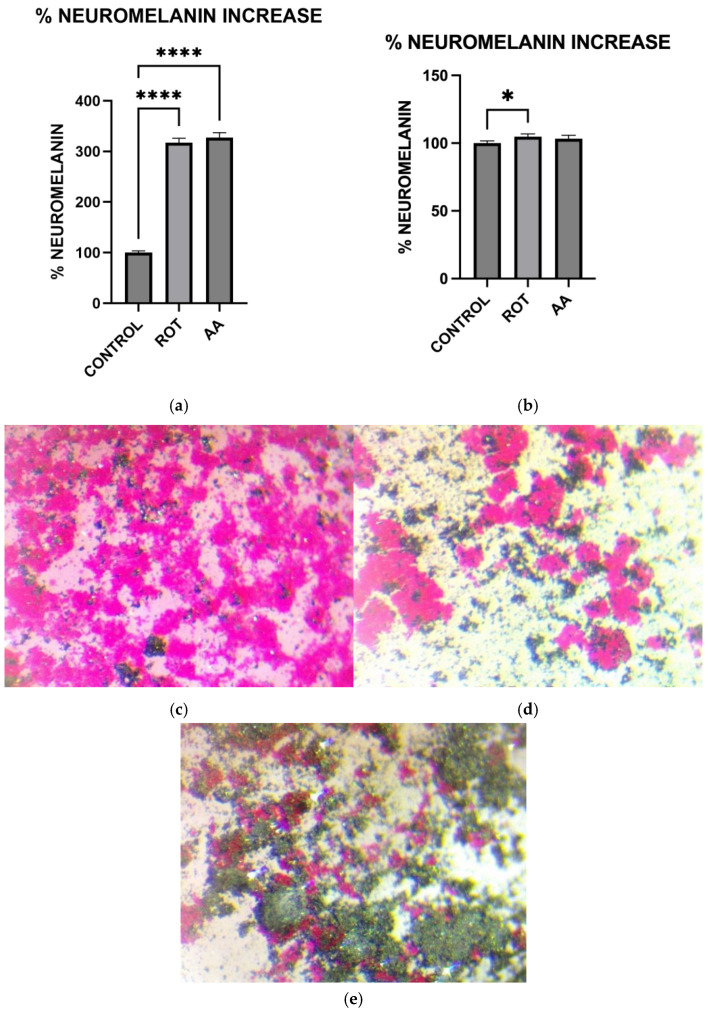
Effect of Toxins on Neuromelanin production in MN9D and HEPG2 cells. (**a**) Neuromelanin levels in MN9D cells. (**b**) Neuromelanin levels in HepG2 cells. (**c**–**e**) Representative brightfield images showing Fontana Masson staining in MN9D cells: (**c**) control, (**d**) rotenone-treated, and (**e**) antimycin A-treated. (Data are represented as mean ± SD (*n* = 5) * indicates difference from control, **** *p* < 0.0001 indicates statistical difference from control by one-way ANOVA).

To investigate the effect of mitochondrial dysfunction on neuromelanin synthesis, MN9D dopaminergic neurons and HepG2 liver cells were treated for 1 h with control buffer (KRB-HEPES pH 7.5), rotenone (1.5 µM), or antimycin A (10 µM), followed by Fontana Masson staining to detect melanin-like pigmentation. In MN9D cells, both rotenone and antimycin A treatments led to a significant increase in neuromelanin accumulation compared to control (Figure 9a). In contrast, HepG2 cells did not show a significant change in melanin levels following treatment with either toxin (Figure 9b). Representative brightfield images showing Fontana Masson staining in MN9D cells: (c) control, (d) rotenone-treated, and (e) antimycin A-treated. Increased dark deposits consistent with elevated neuromelanin production are seen in the representative brightfield images showing Fontana Masson staining in toxin-treated MN9D cells.

### 3.10. Cellular Antixodant Response Was Activated in Dopaminergic Neurons Following Exposure to Toxins

**Figure 10:**
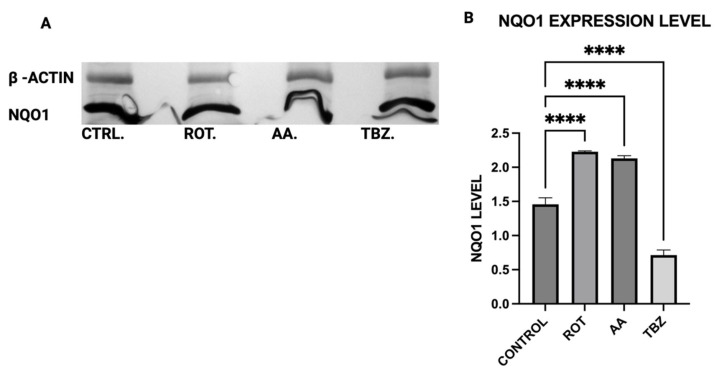
NQO1 protein expression in dopaminergic neurons. (**A**) Representative Western blot bands showing NQO1 expression levels in all treatment groups, (**B**) Quantitative analysis of Western blot bands. (Data are represented as mean ± SD (*n* = 3), **** *p* < 0.0001 indicates statistical difference from control by one-way ANOVA).

To assess the cellular antioxidant response to mitochondrial toxins, the expression of NAD(P)H:quinone oxidoreductase 1 (NQO1), a key oxidative stress response enzyme, was evaluated in MN9D dopaminergic neurons following a 1 h treatment with control buffer (KRB-HEPES pH 7.5), rotenone (1.5 µM), antimycin A (10 µM), or tetrabenazine (1 µM). Quantitative analysis of the Western blot images revealed that both rotenone and antimycin A significantly increased NQO1 expression compared to control (Figure 10B). In contrast, tetrabenazine treatment resulted in a significant decrease in NQO1 levels (Figure 10B).

### 3.11. Effect on Toxins on Cellular Detoxification in Dopaminergic Neurons

**Figure 11:**
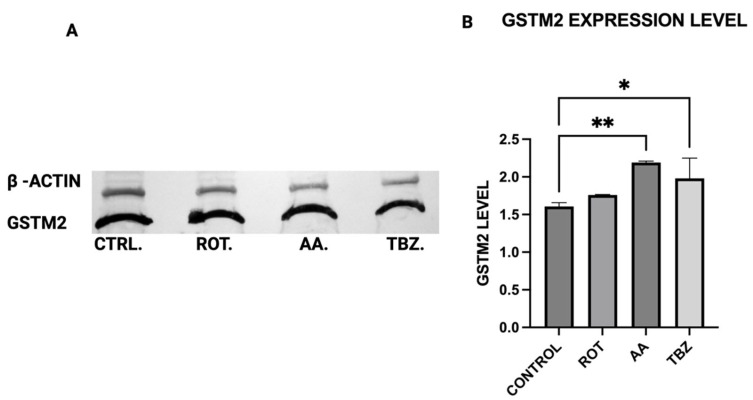
GSTM2 protein expression in dopaminergic neurons. (**A**) Representative Western blot images show GSTM2 protein levels across treatment groups, normalized to β-actin. (**B**) Quantification of Western blot bands (Data are represented as mean ± SD (*n* = 3), * and ** indicates difference from control by one-way ANOVA).

To evaluate the cellular detoxification response to mitochondrial toxin exposure, we assessed the expression of glutathione S-transferase Mu 2 (GSTM2), a key enzyme involved in oxidative stress defense and neuromelanin regulation—in MN9D dopaminergic neurons. Cells were treated for 1 h with control buffer (KRB-HEPES pH 7.5), rotenone (1.5 µM), antimycin A (10 µM), or tetrabenazine (1 µM), followed by Western blot analysis. Quantification of Western blot bands revealed a significant increase in GSTM2 expression in cells treated with antimycin A compared to control (Figure 11B). A non-significant increase was observed in the rotenone group, suggesting a milder or delayed activation of this pathway under Complex I inhibition (Figure 11B). No change in GSTM2 expression was detected in tetrabenazine-treated cells, aligning with its lack of impact on mitochondrial function and oxidative stress markers (Figure 11B).

## 4. Discussion

Our study provides compelling evidence that mitochondrial dysfunction induced by rotenone and antimycin A contributes to dopaminergic neuronal death through energy depletion, which drives oxidative stress, and catecholamine dysregulation. These findings align with previous reports demonstrating that mitochondrial toxins can mimic key pathological features of Parkinson’s disease (PD) [26,27].

Both rotenone (Complex I inhibitor) and antimycin A (Complex III inhibitor) significantly reduced ATP levels in MN9D dopaminergic neurons (Figure 1). The decline in cellular energy production is a well-established consequence of mitochondrial electron transport chain (ETC) inhibition, leading to impaired neuronal function and increased vulnerability to oxidative stress [19]. Previous studies have shown that chronic inhibition of Complex I by rotenone disrupts ATP synthesis, ultimately contributing to neurodegeneration [25]. Our findings support this by demonstrating that acute exposure to rotenone results in immediate ATP depletion, suggesting that failure (or reduction) of efficient metabolic energy production could be one of the earliest triggers of PD-related neurodegeneration.

To further evaluate whether the observed responses were specific to dopaminergic neurons, we included HepG2 liver cells as a non-neuronal control. HepG2 is a widely used human hepatic cell line that possesses functional mitochondria but does not share the same metabolic or neurotransmitter-specific burdens as dopaminergic neurons. This comparative approach allowed us to assess whether the effects of rotenone and antimycin A were selective for energy-intensive neuronal populations. While both toxins induced some level of oxidative stress in HepG2 cells, the effect was significantly lower compared to MN9D cells (Figure 6 and Figure 8). Notably, cell viability in HepG2 cells remained relatively preserved under rotenone treatment, in contrast to the marked loss observed in MN9D cells, reinforcing the idea that dopaminergic neurons are more vulnerable to mitochondrial dysfunction due to their higher metabolic demands [28].

In addition to the biochemical markers assessed in this study, we recognize that mitochondrial morphology including number, density, and fragmentation provides critical insights into the overall status of the mitochondrial network. Mitochondrial fragmentation has been linked to impaired energy production and increased oxidative stress, particularly in neurodegenerative conditions such as Parkinson’s disease. Although our study did not directly assess mitochondrial dynamics, the observed ATP depletion and elevated ROS levels are strongly indicative of disrupted mitochondrial homeostasis. Furthermore, the increased vulnerability of MN9D neurons compared to HepG2 cells suggests that dopaminergic neurons may lack the compensatory capacity to maintain mitochondrial integrity under toxin-induced stress. These findings reinforce the notion that mitochondrial structural alterations are likely involved in the cascade of events leading to neuronal dysfunction and death following exposure to rotenone and antimycin A.

We observed significant catecholamine dysregulation following exposure to rotenone and antimycin A. Interestingly, rotenone-mediated dopamine depletion followed a mechanism distinct from tetrabenazine, (a vesicular monoamine transporter-2 inhibitor). This suggests that mitochondrial dysfunction can directly impact dopamine metabolism, especially storage as previously hypothesized [16]. This observation is also consistent with the proposal [16] that Complex I inhibition mediated reduction in efficient ATP production plays a critical role in disrupting dopamine homeostasis. Furthermore, the differential effects of rotenone and tetrabenazine imply that mitochondrial dysfunction contributes uniquely to dopamine loss is independent of the vesicular monoamine transporter-2 inhibition. Western blot analysis revealed that dopamine depletion after exposure to toxins is not a factor of enzyme expression. Rotenone treatment significantly reduced TH expression (however not affecting DOPA production) while Antimycin A has no effect on TH expression. The implication of this is that depleted dopamine is not due to lack of DOPA, pointing at energy deficit as a key player.

When Dopa Decarboxylase (DDC) expression level was assessed, no significant difference was observed in DDC expression across all treatment groups, implying that the conversion of L-DOPA to dopamine is not directly affected by mitochondrial inhibition or vesicular monoamine transporter blockade, further pointing at the role of energy deficit in dopamine depletion instead.

Rotenone significantly reduced Dopamine Beta-Hydroxylase (DBH) expression, while antimycin A caused a non-significant reduction. This suggests that dopamine conversion to norepinephrine is not a probable cause for its depletion in toxins treated MN9D cells. The results of the ATP assay, Catecholamine measurement and Western blot put together confirm a significant role of energy deficit in catecholamine dysregulation observed in toxins treated cells.

Our results confirm that Complex I inhibition leads to increased cell death specifically in dopaminergic neurons, suggesting that rotenone selectively induces apoptosis in these neurons due to their high metabolic demand and reliance on oxidative phosphorylation. In contrast, HepG2 cells demonstrated higher resistance to rotenone-induced cytotoxicity, again highlighting the selective vulnerability of dopaminergic neurons. The preferential vulnerability of dopaminergic neurons to mitochondrial toxins has been attributed to their extensive axonal arborization, which increases their energy demand [28]. By demonstrating that rotenone and antimycin A exacerbate cell death in MN9D neurons, our study provides additional evidence supporting the role of mitochondrial dysfunction in PD pathogenesis.

Consistent with prior findings, we observed that exposure to mitochondrial inhibitors significantly increased reactive oxygen species (ROS) production in dopaminergic neurons. Oxidative stress is a well-documented contributor to PD pathology, as ROS-mediated damage exacerbates mitochondrial dysfunction and neuronal degeneration [27,29], and our data further demonstrates that this oxidative burden was more pronounced in MN9D cells compared to HepG2 cells. Interestingly, our study also found that neuromelanin production was elevated in response to Complex I and III inhibition. Neuromelanin has been proposed as both a protective mechanism against oxidative stress and a potential contributor to neurotoxicity when excessively accumulated [15]. The accumulation of neuromelanin in response to oxidative stress aligns with previous studies proposing a neuroprotective role for this pigment. Neuromelanin may act as a metal chelator, reducing iron-mediated ROS generation, but excessive accumulation can exacerbate neurotoxicity [15]. Our findings are consistent with reports suggesting that oxidative stress-driven neuromelanin formation serves as an adaptive mechanism in early PD pathology but becomes detrimental under prolonged mitochondrial dysfunction [30]. In contrast, no significant change in melanin content was observed in HepG2 cells following toxin exposure, consistent with their non-catecholaminergic nature. This cell-type specificity underscores the relevance of neuromelanin in PD pathology and supports its use as a marker of dopaminergic stress. Western blot analysis revealed increased GSTM2 expression in antimycin A-treated neurons, with a non-significant increase in the rotenone group, suggesting an adaptive response to oxidative stress and neuromelanin accumulation. Since GSTM2 plays a role in detoxification and neuromelanin homeostasis as discussed in our hypothesis paper [16], its upregulation in mitochondrial-stressed cells may reflect a protective mechanism. NQO1 expression significantly increased in both rotenone and antimycin A groups, indicating a response to heightened oxidative stress and quinone detoxification. This supports previous findings that mitochondrial dysfunction elevates oxidative damage, requiring increased quinone-reducing enzyme activity. Interestingly, tetrabenazine decreased NQO1 expression, suggesting that vesicular monoamine depletion does not trigger the same oxidative stress response as mitochondrial inhibition. Our results suggest that neuromelanin formation may initially serve as a defense mechanism against oxidative damage but could become pathogenic if dysregulated over time.

Our findings have several important implications for understanding PD pathogenesis. First, the differential effects of rotenone and tetrabenazine on dopamine depletion suggest that mitochondrial dysfunction directly contributes to neurotransmitter dysregulation, independent of vesicular transport mechanisms. This underscores the need to explore therapeutic strategies that enhance mitochondrial bioenergetics to prevent dopamine loss. Second, the observed increase in neuromelanin production raises questions about whether targeting neuromelanin metabolism could offer neuroprotective benefits. The inclusion of HepG2 cells in our study allowed us to discern neuron-specific responses to mitochondrial toxins, particularly with regard to oxidative stress, ATP depletion, and neuromelanin formation. These comparisons strengthen the conclusion that dopaminergic neurons possess a unique vulnerability to mitochondrial stress, which may underlie their selective degeneration in PD. One limitation of our study is the use of an in vitro model, which may not fully capture the complexity of in vivo PD pathology. Future research should validate these findings in animal models and human-derived neuronal cultures to confirm their relevance to disease progression. Additionally, investigating whether pharmacological interventions aimed at restoring mitochondrial function can mitigate rotenone- and antimycin A-induced neurotoxicity could provide insights into potential therapeutic strategies.

## 5. Conclusions

In conclusion, our study highlights the critical role of mitochondrial dysfunction in driving dopaminergic neuron degeneration. By elucidating the mechanistic links between mitochondrial energy depletion, oxidative stress, and dopamine dysregulation, we provide valuable insights into the early molecular events contributing to PD. This study demonstrates that mitochondrial Complex I and III inhibition by rotenone and antimycin A leads to ATP depletion, oxidative stress, and catecholamine dysregulation in dopaminergic neurons. These findings further support the role of mitochondrial dysfunction in PD and provide potential avenues for therapeutic intervention targeting mitochondrial resilience.

## Data Availability

The original contributions presented in this study are included in the article, further inquiries can be directed to the corresponding author.

## Abbreviations

The following abbreviations are used in this manuscript:

SNpc	Substantia nigra pars compacta
DA	Dopamine
NM	Neuromelanin
PD	Parkinson’s Disease
MN9D	Mouse Dopaminergic Neuronal Cell line
MTT	3-(4,5-dimethylthiazol-2-yl)-2,5-diphenyltetrazolium bromide
KRB-HEPES	Krebs-Ringer Solution (HEPES Buffered)
SDS	Sodium Dodecyl Sulfate
ATP	Adenosine Triphosphate
TH	Tyrosine Hydroxylase (TH),
DDC	Dopa Decarboxylase (DDC),
DBH	Dopamine β Hydroxylase (DBH),
GSTM2	Glutathione S- transferase Mu 2 (GSTM2)
NQO1	NADPH quinone oxidoreductase 1 (NQO1)
PVDF	Polyvinylidene Fluoride
